# Wings of Discovery: Using *Drosophila* to Decode Hereditary Spastic Paraplegia and Ataxias

**DOI:** 10.3390/cells14181466

**Published:** 2025-09-19

**Authors:** Rachele Vivarelli, Chiara Vantaggiato, Maria Teresa Bassi, Filippo Maria Santorelli, Maria Marchese

**Affiliations:** 1 Department of Neurobiology and Molecular Medicine, IRCCS Fondazione Stella Maris, Calambrone, 56128 Pisa, Italy; rachelevivarelli@gmail.com; 2 Laboratory of Medical Genetics, Scientific Institute IRCCS Eugenio Medea, Bosisio Parini, 23842 Lecco, Italy; chiara.vantaggiato@lanostrafamiglia.it (C.V.); mariateresa.bassi@lanostrafamiglia.it (M.T.B.)

**Keywords:** *Drosophila melanogaster*, hereditary spastic paraplegia, hereditary ataxias, Disease modeling

## Abstract

Hereditary spastic paraplegia (HSP) and hereditary ataxias (HA) are clinically and genetically heterogeneous neurodegenerative disorders that primarily affect motor coordination and neural integrity. Despite distinct pathological features, such as pyramidal tract degeneration in HSP and spinocerebellar pathway involvement in HA, these conditions share overlapping genetic pathways and mechanisms. The fruit fly *Drosophila melanogaster* has emerged as a powerful model organism for investigating the molecular basis of rare diseases, including HSP and HA. Its genetic tractability, rapid life cycle, and high degree of gene conservation with humans make it a cost-effective and ethically viable platform for disease modelling. In this review, we provide a comprehensive overview of *Drosophila*-based models for HSP and HA. We highlight the use of advanced genetic tools, including RNA interference, CRISPR/Cas9, and the GAL4/UAS system, as well as behavioral and neuroanatomical assays to model disease features. Furthermore, we discuss the application of genetic “avatars” and high-throughput drug screening platforms to test therapeutic candidates. Collectively, these models have deepened our understanding of the pathophysiology of HSP and HA, offering valuable insights for the development of targeted therapies and approaches to personalized medicine.

## 1. Introduction

**Figure 1:**
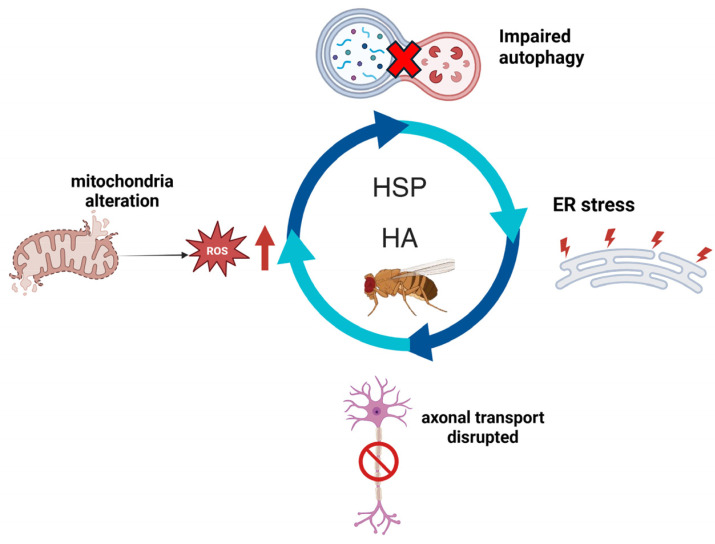
The figure shows the main molecular pathways shared by HSP and HA and conserved in *Drosophila* model. Created with Biorender.com.

The term hereditary spastic paraplegia (HSP) refers to a clinically and genetically heterogeneous group of neurodegenerative disorders characterized by progressive spasticity and weakness of the lower limbs [1]. Most patients exhibit the same clinical features, including bilateral spasticity of the legs, particularly evident while walking, muscle weakness, hypertone in the lower limbs, hyperreflexia, and a bilateral Babinski sign [2]. Post-mortem analyses conducted on patient tissues have revealed the presence of axonal degeneration that begins at the distal end and slowly progresses toward the cell body; this phenomenon is why these conditions are often referred to as “dying-back” axonopathies [3]. In complicated forms, alterations may also affect other brain structures, such as the cerebellum, cerebral cortex, basal ganglia, and white matter [4]. Based on symptomatology HSP can be classified as either pure or complicated. It is defined as pure if these symptoms represent the only observed clinical manifestations, whereas it is considered complicated when additional neurological or extra-neurological symptoms are present. These may include cognitive/mental impairment, cerebellar ataxia, peripheral neuropathy, epilepsy, optic atrophy, retinal alterations, cataracts, dystonia, and hypokinetic movement disorders [5]. The current genetic classification of the different forms of HSP is based on the type of inheritance, chromosomal locus, and the responsible mutation. HSP can be inherited in an autosomal dominant (AD), autosomal recessive (AR), X-linked, or maternal (mitochondrial) pattern [1]. The prevalence of AD HSP has been reported to range between 0.5 and 5.5 cases per 100,000 individuals. In contrast, AR HSP prevalence varies from 0.3 to 5.3 per 100,000 in hospital-based cohorts, and between 0.6 and 2.6 per 100,000 in studies incorporating multiple data sources. X-linked and mitochondrial forms of HSP are relatively rare and are predominantly associated with congenital presentations [6]. HSP and hereditary ataxia (HA) share overlapping clinical and genetic features, as both involve progressive neurodegeneration affecting motor pathways including the corticospinal tracts and cerebellum; furthermore, certain genes can cause phenotypes spanning from spastic paraplegia to cerebellar ataxia, reflecting a disease spectrum with shared molecular mechanisms (Figure 1). However, in HSP there is degeneration of the pyramidal tract, while in HA there is progressive degeneration of the spinocerebellar tract and Purkinje cells [7]. Furthermore, HSP and HA share pathological mechanisms and cellular pathways [8].

**Figure 2:**
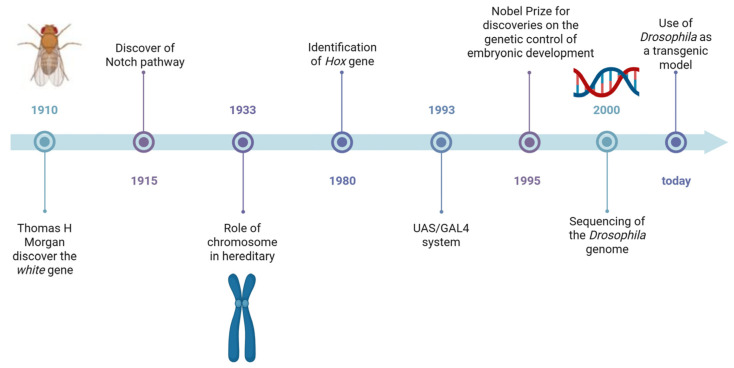
Timeline of major discoveries made using *Drosophila* as animal model. Created with Biorender.com.

Regarding main features in HA, patients show a progressive loss of coordination and movement ability, as well as limited eye movement [9]. The classification of HA is based on the mode of transmission and is divided into autosomal dominant cerebellar ataxia, autosomal recessive, X-linked HA, and ataxias with mitochondrial gene mutations [10]. The AD forms are characterized by trinucleotide repeat expansions, the most common being the CAG triplet expansions within the coding region of the gene [9]. Among the AR forms, Friedreich’s ataxia (FRDA) is the most common, with a population frequency of 1–2:50,000 [9,10,11]. Patients with the X-linked form present with early onset, dysmetria, and dysdiadochokinesia. Progressive forms of ataxia can also be often associated with mitochondrial dysfunction [12]. The *Drosophila melanogaster* model is a powerful tool to study genetically and clinically heterogeneous disorders. It is widely used to investigate rare human diseases and to assess the pathogenicity of specific genetic variants [13]. *Drosophila* is a small fly belonging to the order *Diptera* and the family *Drosophilidae*, commonly known as the fruit fly [14]. This small invertebrate has led to many important discoveries in the field of biology over the years, and it is still used today as a model organism in over 1800 laboratories worldwide, thanks to numerous seminal work that make it ideal for research (Figure 2). It is small, easy to handle, and cost-effective to maintain in the laboratory [14]. It has a short lifespan, a high reproductive capacity, and an external development, allowing researchers to monitor each stage of its growth. Additionally, its genome has been fully sequenced, and homologs have been identified by nearly 75% of genes associated with human diseases. With the combination of genome-wide genetic screening, advanced sequencing techniques (such as RNA-seq and ChIP-seq), and metabolomics analyses, *Drosophila* has become a widely used model for studying human diseases, including those affecting the central and peripheral nervous systems [15]. Despite significant differences in the overall anatomy of *Drosophila* and human brains, they share many conserved genetic, cellular, electrophysiological, and chemical characteristics. Like vertebrates, fruit flies rely on a diverse range of neuronal types to process information [16].

## 2. Modelling Diseases in *Drosophila*

**Figure 3:**
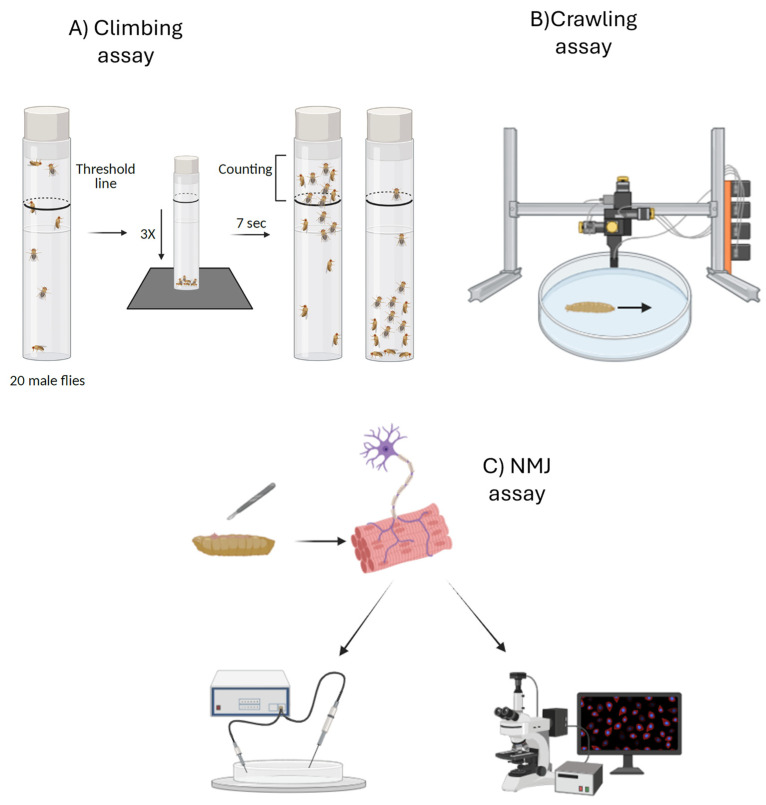
The figure shows three alternative techniques that can be used in *Drosophila* models for HA and HSP. From left to right, we see the climbing assay (**A**), a behavioral test used to assess motor function by evaluating the flies’ ability to climb up a test tube over time [20]. The crawling assay is another behavioral test, performed on larvae (**B**), where the animal’s movement across a Petri dish is monitored [21]. Finally, the NMJ assay (**C**) is a morphological and functional test in which the larva is dissected, the neuromuscular junction is isolated, and immunofluorescence or electrophysiological analyses can be performed on it [22]. All these tests allow the assessment of motor function and the detection of early alterations in ataxia and paraparesis models. Created with Biorender.com.

Two alternative techniques can be used to study diseases like HA and HSP in *Drosophila*: forward genetics and reverse genetics. The term forward genetics refers to a technique that involves generating random mutations, with flies then identified by analyzing their phenotype [16]. These mutations can be induced using chemical or insertional mutagenesis techniques [17]. Reverse genetics begins with a gene of interest (such as a *Drosophila* homolog of a human disease gene) and tests its function by disrupting or modifying it. Unlike forward genetics, which proceed from phenotype to gene, reverse genetics follow the opposite logic, moving from gene to phenotype. A variety of genetic tools are available to manipulate gene function in Drosophila [15]. One widely used approach is the CRISPR/Cas9 system, which enables targeted gene knockout or the introduction of loss-of-function mutations, thereby allowing replication of causative variants identified in human disorders. Alternatively, researchers can employ transposon-mediated mutagenesis or excision of existing transposable elements. Another key methodology is the GAL4-UAS system, a binary expression tool that permits spatial and temporal control of transgene expression. This system can be employed to ectopically express either wild-type or mutant genes, or to drive RNA interference (RNAi) constructs placed under the UAS promoter. In the latter case, GAL4-driven expression of UAS-RNAi transgenes results in gene silencing and facilitates the generation of loss-of-function phenotypes. Such RNAi-mediated downregulation has been extensively used to model HSPs in *Drosophila*, as it enables the reduction in target gene expression in specific tissues or developmental stages [18]. Similarly, models of HAs often exploit the GAL4-UAS system to achieve either RNAi-mediated knockdown or the overexpression of mutant proteins, thereby recapitulating both loss-of-function and gain-of-function mechanisms [19]. Together, these complementary genetic strategies provide powerful means to model disease phenotypes in vivo and to investigate the molecular and cellular consequences of gene inactivation or toxic protein expression. These models exhibit various phenotypes, including motor and locomotion deficits. They also show alterations at the neuromuscular junction (NMJ), such as changes in synaptic bouton number, size, and branching, as well as functional impairments in synaptic transmissions. These motor alterations can be easily analyzed in *Drosophila* using techniques that assess movement ability, such as the climbing assay, crawling assay, and NMJ assay. In the case of the climbing assay, adult flies are used; they are placed in a tube, gently tapped to the bottom, and their ability to climb up the walls above a mark (usually at 6 cm from the bottom) within a defined time interval (usually 8–10 s) is measured. A reduction in performance is indicative of motor deficits, neurodegeneration, neuromuscular dysfunctions, or aging (Figure 3) [20]. The crawling assay, on the other hand, is used to analyze the locomotion of larvae, typically at the third instar stage. In this test, larvae are placed on a flat surface (such as an agar-coated Petri dish), and their movement is observed or recorded over a defined period. This assay is particularly useful for identifying early motor alterations caused by genetic mutations (Figure 3) [21]. Another test that can be employed in these models is the NMJ assay, a combination of morphological and functional analyses aimed at assessing NMJ integrity. Morphological evaluation involves dissection, fixation, and immunostaining of larval or adult tissues using synaptic markers, followed by fluorescence microscopy to quantify synaptic bouton number, size, and branching, while functional assessment can include electrophysiological recordings of synaptic transmission (Figure 3) [22]. Together, these approaches provide a comprehensive evaluation of synaptic structure and function, essential for understanding neuromuscular deficits in HA and HSP models [23].

These models are crucial for understanding the mechanisms underlying neurodegeneration in HSP and HA and provide insights for therapeutic development. In this review, we examine and illustrate the different *Drosophila* models generated for various forms of HSP and HA, highlighting the genetic strategies used to recreate disease-relevant phenotypes, the observed phenotypic manifestations, and the insights gained into underlying pathogenic mechanisms [24]. Additionally, we discuss how these models have been employed to identify genetic modifiers and potential therapeutic targets, emphasizing the versatility and power of *Drosophila* as a tool for studying neurodegenerative disorders. 

## 3. Investigating Visual Dysfunction in Drosophila Models of HA and HSP

Patients affected by HA and HSP may present significant visual impairments, although the frequency and severity of these manifestations vary widely depending on the genetic subtype. Among ataxias, some of the forms most frequently associated with visual deficits include spinocerebellar ataxia 7 (SCA7), FRDA, and to a lesser extent, SCA1, SCA3, and SCA6. SCA7 is notably associated with pigmentary retinopathy and progressive macular degeneration, which can lead to complete blindness in patients, often preceding the onset of cerebellar motor symptoms [25]. Visual disturbances have also been described in SCA1 and SCA3, where defects in visual synaptic transmission, photoreceptor degeneration, and reduced retinal integrity have been reported. In FRDA, an autosomal recessive disorder caused by mutations in the *FXN* gene, optic atrophy, color vision deficits, and abnormalities in visual evoked potentials have been documented, suggesting impaired conduction along the optic nerve and visual cortex [26]. In fact, certain forms of HSP in patients, particularly those linked to mutations in *SPG7*, *SPG11*, and *SPG15*, also present with optic atrophy and retinal abnormalities, including reduced visual acuity, optic disc pallor, and in some cases, cortical blindness. The *Drosophila* eye has a modular organization; the retina consists of approximately 750 ommatidia, each separated from its neighbor and containing eight distinct types of photoreceptors [27]. This architecture allows for easy detection of morphological changes induced by genetic mutations, such as cellular disorganization, apoptosis, or neuro-axonal degeneration. Furthermore, although it is not part of the central nervous system, the eye is innervated, enabling its use as an efficient platform for both genetic and pharmacological screening [28]. Visual alterations are observed in many *Drosophila* models replicating forms of HA and HSP. In the *Drosophila* model of *sca7*, expression of expanded human *ATXN7* induces retinal degeneration, neuronal apoptosis, ommatidial disorganization, and reduced electrophysiological response to light [19]. Similarly, degenerative ocular phenotypes, including the so-called “rough eye” phenotypes, have been observed in models of *sca3, sca13, sca1*, and *sca31*, reflecting neuronal dysfunction in the retina and altered visual signal transduction [29,30,31]. As for HSP, visual impairments are especially evident in *spg7* and *spg4* models. In *spg7*, photoreceptor synaptic terminals were found to be disorganized and displayed accumulations of swollen and morphologically abnormal mitochondria [32]. The *spg4* model also shows significant visual alterations, such as defective ommatidial morphology and reduced eye size [33].

These findings underscore the importance of the visual system as a vulnerable and clinically relevant target in various forms of HA and HSP. The use of *Drosophila melanogaster* as a model organism, particularly through eye-specific assays, proves to be a powerful tool to dissect the molecular mechanisms underlying visual neurodegeneration

## 4. *Drosophila* in the Study of HSP Disorders

A total of 18 orthologous *SPG* genes have been identified in *Drosophila*, including 7 dominant and 11 recessive forms (https://flybase.org/lists/FBhh/; accesed on 17 June 2025) (Table 1). *Drosophila melanogaster* models of HSP have revolutionized our understanding of the cellular and molecular mechanisms of these rare neurodegenerative disorders, which affect the central nervous system, primarily motor neurons and spinocerebellar nerve fibres. Among the most studied and common forms are the genes *SPAST* (SPG4), *ATLASTIN* (SPG3A) and *SPG7* [34].

Many of these genes encode proteins involved in intracellular trafficking of proteins and organelles. The human *SPAST* gene encodes spastin, an ATP-dependent microtubule severing protein and shares sequence similarity with the N-terminal microtubule interacting and trafficking domain of the protein associated with SPG20 [35]. The protein spastin plays a critical role in ER-to-Golgi membrane trafficking and in the completion of cytokinetic abscission, and evidence further suggests its involvement in axonal outgrowth and branching [35]. There is a single fly orthologue of *SPAST*, *spas*, which has been studied to better understand the role of spastin in neurodegeneration. Loss of *spas* function in the fly nervous system is not lethal, but affected flies progressively develop movement defects until they become completely immobile. Neurodegeneration is visible in the fly brain, with a clear presence of neurons undergoing apoptosis, these features being reminiscent of those observed in HSP patients [36]. In addition to knockdown analyses, overexpression of human wildtype and mutant *SPAST* was also performed in flies (Table 1). There is a functional equivalence between human and fly genes, suggesting conserved functionality between the two species [37]. An additional frequent form is SPG3A caused by mutations in the *ATL1* gene, which encodes atlastin, a dynamin-related GTPase that plays a role in formation of the ER network and in axon elongation in neurons [38]. In *Drosophila*, *atlastin* knockdown mediated by RNAi leads to loss of *atlastin* in neurons and ensuing ER morphological defects (Table 1) [39], synaptic dysfunction, and microtubule disorganization. It has been shown that both downregulation and overexpression of *atlastin* in *Drosophila* motor neurons lead to impaired locomotion. Larvae exhibit reduced crawling speed and contraction frequency, while adult flies show a progressive decline in climbing ability highlighting the critical role of this protein in neuronal health [40]. Among the most common HSP recessive forms, we list *SPG7*, encoding paraplegin, a component of the m-AAA protease, an ATP-dependent proteolytic complex of the mitochondrial inner membrane and the protein has roles in diverse cellular processes including membrane trafficking, intracellular motility, organelle biogenesis, protein folding, and proteolysis [41]. In *Drosophila* there is a single orthologue, *spg7* [32]. To generate a knock-out *spg7* fruit fly model, the CRISPR/Cas9 gene editing technique has been adopted. The mutant flies showed a shorter lifespan, motor impairment, neuronal and muscular degeneration, and finally the presence of dysmorphic mitochondria, indicating that the phenotypes of the *spg7* mutants are associated with mitochondrial dysfunction (Table 1) [32]. SPG11 CRISPR/Cas9 and SPG15 RNAi *Drosophila* knockdown models present autophagosome accumulation, enlarged lysosomes, a reduced number of free lysosomes, autophagic lysosomal reformation defects in larval muscles, and locomotor deficits [42]. *SPG11* and *SPG15* encode proteins that are essential for the proper functioning of the autophagy-lysosome pathway. SPG11 is involved in the formation and trafficking of autolysosomes and in maintaining lysosomal homeostasis, while SPG15 plays a key role in autophagic lysosomal reformation and the maturation of lysosomes. The loss of either protein disrupts lysosomal clearance and autophagic flux, leading to the accumulation of autophagosomes and impaired muscle function, which in turn contributes to locomotor deficits observed in the fly models. Moreover, the specific function of reticulon-2 (RTN2) has been most clearly defined in *Drosophila*, making this model a paradigmatic exemplar for the field. Espadas et al. demonstrated that *Drosophila* reticulon drives dynamic constriction and fission of ER membranes, establishing a mechanistic basis for reticulon-dependent ER remodeling [43]. Building on this, Pérez-Moreno et al. showed that the *Drosophila SPG12/RTN2* orthologue, reticulon-like 1 (Rtnl1), governs presynaptic ER organization and Ca^2+^ dynamics, directly linking reticulon activity to neuronal physiology [44]. Together, these studies position *Drosophila* as the key reference system for understanding RTN2/reticulon function in ER dynamics, and for other HSPs proteins involved in ER network.

**Table 1:** Summary table of HSP/SPG genes relevant to *Drosophila* research, including conserved orthologues and loci without orthologue, the genetic models developed, methodologies employed, and main phenotypes observed.

Human Genes	Protein Function	Identity and Similarity	*Drosophila* Genes	Type of Model	Behavior	Morphology	References
SPG3A/*ATL1*	Membrane-anchored dynamin-like GTPase mediating GTP-dependent fusion of ER membranes; essential for maintaining a continuous tubular ER network and axonal homeostasis	52–57% and 71–77%	*atlastin*	Loss of function	reduce climbing performance	muscular ER fragmentation at the NMJs, and progressive degeneration of dopaminergic neurons	[45,46,47,48]
SPG4/*SPAST*	ATP-dependent microtubule severing enzyme that preferentially cuts polyglutamylated microtubules; regulates axonal microtubule dynamics and transport	44% and 55%	*spas*	Loss of function and gain of function	adults have severe movement defects, cannot fly, and have weak legs	larvae have altered NMJs in which presynaptic boutons are more numerous and smaller than in wild type	[36,49,50,51,52]
*SPG7*	Catalytic component of the m-AAA protease, a protease that plays a key role in proteostasis of inner mitochondrial membrane proteins, and which is essential for axonal and neuron development	58% and 75%	*spg7*	Loss of function	shortened lifespan, climbing or flight defects, sensitivity to stressors	photoreceptor synaptic terminal disorganized, degeneration of indirect flight muscle and mitochondrial trafficking defects	[32]
SPG10/*KIF5A*	Microtubule-dependent motor required for slow axonal transport of neurofilament proteins	60% and 76%	*khc*	Gain of function	complete paralysis, reduced lifespan, no stable flight	axons innervating posterior segments are considerably longer	[53,54,55]
SPG11	play a role in neurite plasticity by maintaining cytoskeleton stability and regulating synaptic vesicle transport		*spg11*	Loss of function	locomotor deficit	autophagosome accumulation, enlarged lysosomes, reduced free lysosomes, autophagic reformation defects	[43]
SPG12/*RTN2*	ER-shaping protein of the reticulon family; regulates curvature of ER tubules and ER network homeostasis	30% and 46%	*rtnl1/rtnl2*	Loss of function	locomotion impairment	ER stress response, abnormalities of ER marker, MT cytoskeleton and mitochondria	[43,44,56]
SPG15/*ZFYVE26*	Phosphatidylinositol 3-phosphate-binding protein required for the abscission step in cytokinesis: recruited to the midbody during cytokinesis and acts as a regulator of abscission. May also be required for efficient homologous recombination DNA double-strand break repair	30% and 50%	*sptz*	Loss of function	locomotor deficit	autophagosome accumulation, enlarged lysosomes, reduced free lysosomes, autophagic reformation defects	[57]
SPG17/*BSCL2*	Plays a crucial role in the formation of lipid droplets (LDs) which are storage organelles at the center of lipid and energy homeostasis	31% and 51%	*seipin*	Loss of function	reduced locomotor activity	mild triacylglycerol storage phenotype	[58]
*SPG20/SPART*	Lipophagy receptor that plays an important role in lipid droplet (LD) turnover in motor neurons	25% and 40%	*spartin*	Loss of function	locomotion impairment	neurodegeneration, progressive vacuolization in adult brain, reduced neurotransmitter release	[59]
SPG30/*KIF1A*	Kinesin motor with a plus-end-directed microtubule motor activity	-	no orthologue	Loss of function	embryos are paralyzed and fail to hatch	nerve outgrowth fails, synaptic bouton defects, loss of SVs and AZs at NMJ	[60,61]
SPG31/*REEP1*	ER membrane protein linking ER tubules to microtubules; required for ER shaping, remodeling, and axonal maintenance	50% and 70%	*reepA*	Loss of function	locomotor dysfunction, shortened lifespan	expansion of ER sheet-like structures	[62,63]
SPG35/*FA2H*	Catalyzes the hydroxylation of free fatty acids at the C-2 position to produce 2-hydroxy fatty acids, which are building blocks of sphingolipids and glycosphingolipids common in neural tissue and epidermis	60% and 75%	*fa2h*	Loss of function	flying disability, behavioral abnormalities	mitochondrial dynamics and autophagy alterations	[64]
SPG39/*PNPLA6*	Catalyzes the hydrolysis of several naturally occurring membrane-associated lipids	45% and 65%	*sws*	Loss of function	progressive behavioral defects	neurodegeneration with vacuole formation	[65,66,67]
SPG61/*ARL6IP1*	Positively regulates SLC1A1/EAAC1-mediated glutamate transport by increasing its affinity for glutamate in a PKC activity-dependent manner	29% and 61%	*arl6IP1*	Loss of function	significant locomotor deficit	ER and mitochondrial disorganization, disrupted lipid droplets	[68,69]
SPG76/*CAPN1*	Calcium-regulated non-lysosomal thiol-protease which catalyzes limited proteolysis of substrates involved in cytoskeletal remodeling and signal transduction	45% and 64%	*calpA/caplB*	Loss of function	locomotor defects	axonal abnormalities	[70]
SPG77/*FARS2*	Is responsible for the charging of tRNA (Phe) with phenylalanine in mitochondrial translation	65% AND 80%	*pheRS-m*	knockout	developmental delay, seizures	mitochondrial tRNAphe and oxidative phosphorylation defects	[71,72]
SPG78/*ATP13A2*	ATPase which acts as a lysosomal polyamine exporter with high affinity for spermine	-	no orthologue	knockdown			[73]
SPG92/*FICD*	Protein that can both mediate the addition of adenosine 5’-monophosphate (AMP) to specific residues of target proteins (AMPylation), and the removal of the same modification from target proteins (de-AMPylation), depending on the context	35% and 60%	*fic*	knockout			[74]

## 5. Genetic Modelling of HA in *Drosophila*

A significant number of SCAs are caused by abnormal expansions of CAG triplet repeats in their respective genes, resulting in the production of proteins containing expanded polyglutamine (polyQ) tracts. These expansions promote the formation of toxic intracellular aggregates, alterations in nucleocytoplasmic trafficking, transcriptional dysregulation, and progressive loss of neuronal function [75]. To understand the molecular mechanisms underlying these diseases and to test potential therapeutic strategies, the use of genetically tractable experimental models is essential. In this context, *Drosophila melanogaster* has emerged as a highly valuable model system due to its well-defined genetics, considerable homology with human genes implicated in SCAs, and the availability of powerful genetic tools that enable detailed analysis of the molecular pathways involved in neurodegeneration [75]. Despite lacking a cerebellar structure homologous to that of vertebrates, *Drosophila* has a complex central nervous system with well-characterized neural circuits and quantifiable motor behaviors that are affected by neurodegeneration. Thanks to the evolutionary conservation of many human genes, the availability of high-throughput genetic tools, and the feasibility of large-scale experiments, *Drosophila* has been widely used to develop transgenic models of various forms of HA [76]. Approximately 22 orthologous genes have been identified in *Drosophila*, including 11 dominant and 11 recessive forms (https://flybase.org/lists/FBhh/) (Table 2). The most common dominant ataxias include SCA1, SCA2, SCA3, SCA6, and SCA7, while the most studied recessive forms include FRDA and ataxia-telangiectasia (AT) [9]. One of the first *Drosophila* models developed to study SCA1 was based on the expression of human *ATXN1* [82Q] and [30Q] using the UAS-GAL4 system; the protein ataxin1 binds RNA, associates with large protein complexes, and interacts with a vast network of proteins and is thought to be involved in transcriptional repression and to regulate Notch and Capicua [77]. This model is known for reproducing pathological features such as nuclear inclusions, retinal degeneration, and impaired locomotion [78]. Notably, neurotoxicity is polyQ-length dependent, with a marked decline in locomotor ability observed as the flies age. The reduced lifespan of these flies further highlights the progressive nature of the disease. In the case of SCA2, the model developed by Lessing and Bonini, induced protein aggregation and neurodegeneration by expressing mutant *ATXN2* in *Drosophila* neurons, where ATXN2 functions as an RNA-binding protein involved in stress granule assembly and the regulation of RNA metabolism [79,80]. Motor dysfunction was a consistent finding, accompanied by reduced climbing ability as the primary behavioral phenotype. Additionally, reduced lifespan was observed, reflecting the neurodegenerative progression in both the nervous system and muscle tissue. For SCA3, *Drosophila* models expressing the C-terminal fragment of human *ATXN3* with 78 [81] or 82 CAG repeats [82] led to retinal degeneration, abnormal eye morphology, and neuronal death. These flies showed reduced motility and exhibited the well-known “rough eye” phenotype, with locomotor dysfunction and loss of climbing ability as further key phenotypic features. ATXN3 is a deubiquitinase that normally participates in protein quality control and ubiquitin-mediated proteostasis, and its polyglutamine-expanded form disrupts these processes, thereby promoting neuronal toxicity. Regarding FRDA, *Drosophila* models with RNAi-mediated downregulation of the frataxin homolog (*fh*) have demonstrated mitochondrial dysfunction, oxidative stress, and impaired locomotion [83,84]. Consistent with these findings, frataxin is a nuclear-encoded mitochondrial iron chaperone that plays a key role in iron-sulfur cluster biogenesis and heme biosynthesis, processes whose disruption underlies the observed defects. These models typically show reduced climbing ability and a shortened lifespan, mirroring the clinical presentation of FRDA, which is closely tied to mitochondrial and neuronal damage. The ATM protein is a member of the phosphatidylinositol 3-kinase family and responds to DNA damage by phosphorylating key substrates involved in DNA repair and cell cycle control. Consistent with this function, *Drosophila* models of AT targeting the *ATM* homolog *tefu* exhibit neurodevelopmental defects and neuronal degeneration [85,86], along with reduced lifespan, increased sensitivity to DNA damage, and impaired repair pathways, thereby recapitulating key aspects of the human disease.

In summary, across these various models, common behavioral traits such as impaired locomotion and shortened lifespan are consistently observed. In addition, many of these models share neurological and mitochondrial defects, such as reduced climbing, and oxidative stress sensitivity, all of which provide valuable insights into the molecular and cellular underpinnings of these diseases.

## 6. Commonalities and Differences

Across the spectrum of SCA and autosomal recessive cerebellar ataxia (ARCA) models, several shared phenotypic and molecular characteristics have been observed. Locomotor deficits and shortened lifespan are among the most consistently reported behavioral phenotypes. These impairments, evident in models of SCA1 (*Atx-1*), SCA2 (*Atx-2*), SCA17 (*Tbp*), and recessive forms like FRDA (*fh*) and SCAR25 (*Atg5*), indicate broad neurodegenerative processes affecting motor coordination and survival [78,83,89,106,123]. Neurodegeneration, particularly in the eye and brain, is a hallmark of many of these models, such as SCA3, SCA7, and dentatorubral-pallidoluysian atrophy (DRPLA), where retinal and neural tissue show progressive collapse, vacuolization, or loss of structure [82,100,114]. In models like SCA5 and SCA12, synaptic and axonal transport defects have also been documented, often accompanying reduced synaptic terminal growth and mitochondrial abnormalities [94,95,96,97,98,99,100,101,102,103]. These synaptic changes often mirror findings in patients and emphasize the utility of *Drosophila* in identifying disease-related cellular phenotypes. At the cellular and molecular levels, mitochondrial dysfunction and oxidative stress sensitivity are commonly observed, where mutations disrupt mitochondrial dynamics, energy metabolism, and autophagy pathways [115,119,123]. These alterations frequently coincide with ER stress, altered cytoskeletal organization, and impaired organelle transport. Such convergence on mitochondrial and ER dysfunction points to their central role in the pathogenesis of ataxias. Some ataxia models reveal developmental abnormalities and failure to hatch, especially in early-onset or severe phenotypes like those of SCAR21 (*yata*) and SCA12 (*tws*), reflecting disruptions in neuronal development or apoptosis pathways [103,120]. Finally, defects in NMJ morphology and function, such as reduced synaptic branching and altered plasticity, are reported in SCAR24, SCAN3, and SETX models, offering insight into motor system vulnerability in ataxias [122,128,129]. *Drosophila melanogaster* models of HSPs reveal numerous shared phenotypic and cellular features that mirror the neurological impairments observed in human patients. A common behavioral hallmark is locomotor impairment, reported across most models [43,44,45,46], frequently accompanied by reduced climbing or flight ability, paralysis, or decreased lifespan. Morphologically, a consistent finding is NMJ disruption, as seen in models like *spas*, *spg7*, *spartin* (SPG20) [32,49,59]. Another prevalent feature is ER and mitochondrial dysfunction, often accompanied by oxidative stress, this is particularly evident in models of *atl* (SPG3A), *rtnl1/rtnl2* (SPG12), *reepA* (SPG31), *fa2h* (SPG35), *arl6IP1* (SPG61), and *fic* (SPG92) [45,46,56,62,64,68,74]. Mitochondrial dynamics, autophagy defects, and ER stress responses are recurring pathological signatures, such as autophagosome accumulation, lysosomal enlargement, and impaired organelle reformation. Some models, such as *pheRS-m* (SPG77), even display early developmental abnormalities and seizure-like activity, reflecting mitochondrial translation and oxidative phosphorylation defects [71].

In summary, despite the genetic and clinical heterogeneity of HA and HSP, *Drosophila melanogaster* models have uncovered a set of strikingly convergent phenotypic and molecular features. Locomotor impairment is a unifying behavioral hallmark across both disease groups, reflecting widespread dysfunction of motor circuits. Neurodegeneration, particularly affecting the eye, brain, and NMJ, emerges as a shared pathological outcome, often accompanied by disrupted synaptic integrity and axonal transport. At the subcellular level, mitochondrial dysfunction, oxidative stress, and ER stress are consistently observed across many models, indicating that defects in energy metabolism and organelle homeostasis are central drivers of neurodegeneration in both HA and HSP. Moreover, early developmental defects and failure to hatch in more severe or early-onset forms highlight the impact of these mutations on neurodevelopmental pathways. These commonalities underscore the value of *Drosophila* as a cross-disease platform, not only for dissecting shared pathogenic mechanisms but also for identifying potential therapeutic targets that may benefit a broad spectrum of hereditary neurodegenerative disorders.

## 7. Conclusions and Future Perspective

For many years, researchers have used fruit flies to explore how developmental signaling pathways work and to better understand the roles of genes linked to human diseases [16], including rare neurodegenerative disorders such as HSP and HA. Reaching a diagnosis and proposing treatments for patients affected by rare and ultra-rare diseases is particularly challenging [130]. In this context, *Drosophila* models offer important advantages, including genetic tractability, short life cycle and the possibility to investigate disease mechanisms in a whole-organism context. These features enable the development of innovative research strategies for studying conditions that are often difficult to address using cell cultures or vertebrate models, due to limitations in complexity, ethical concerns, and longer experimental times [131]. Many genetic techniques were originally developed in the fruit fly and later adapted for use in mammalian model systems, for example, techniques to introduce transgenes into the genome using pronuclear injection or viral-mediated transgenesis [132,133], as well as the Gal4/UAS system, RNAi, and CRISPR/Cas9. These techniques are key tools for “humanizing” *Drosophila*, allowing researchers to replicate human pathological mutations and study rare variants of human proteins [134,135]. By taking advantage of these available genetic tools, researchers have been able to model and characterize many forms of both HSP and HA, enabling the identification of disease-causing genes, the investigation of molecular mechanisms, and the establishment of genotype–phenotype correlations.

The use of *Drosophila* has certain limitations. Although flies do not possess human-like cognitive abilities, they can perform some complex behaviors, including learning and memory. Additionally, despite a high degree of gene conservation, approximately 40% of human genes are either absent in *Drosophila* or perform different functions, which can restrict the modeling of certain disease mechanisms.

Anatomical differences between humans and flies, such as the absence of a cerebral cortex, myelinated neurons, and a cerebellum, further limit the study of processes that rely on these structures. Similarly, variations in cellular composition, organ complexity, and physiology may influence the manifestation of phenotypes and responses to pharmacological treatments, potentially reducing translational relevance [130]. Furthermore, some pathways or disease processes that involve higher-order cognitive functions or specific tissue interactions cannot be fully replicated in flies. Despite these constraints, *Drosophila* remains a powerful model for investigating conserved molecular pathways, genetic interactions, and basic cellular mechanisms underlying human diseases [130]. In *Drosophila*, wrapping glia plays a crucial role in ensuring proper neuronal signaling. They regulate axon diameter and conduction speed, and their differentiation depends on gap junctions and FGF signaling. Importantly, beyond influencing conduction speed, wrapping glia are essential for the precision of neuronal signaling and coordinated locomotor patterns. These findings highlight how glial cells actively shape neuronal communication, an aspect relevant to understanding glial contributions to neurodegenerative and neuromuscular disorders [136]. These limitations can be overcome by employing murine models, which possess a fully myelinated central nervous system and brain structures homologous to those in humans. In mice, behavioral and neuropathological phenotypes such as spasticity, tremors, and motor coordination deficits can be accurately reproduced, allowing for detailed functional validation of disease genes and therapeutic interventions [131]. Despite these challenges, numerous studies have demonstrated that drug responses in *Drosophila* models often parallel those observed in humans, particularly in neurodegenerative and oncological diseases, highlighting their utility as a cost-effective and rapid screening platform [134]. To further enhance translational relevance, human cellular models, especially patient-derived induced pluripotent stem cells (iPSCs), are employed to study patient-specific phenotypes such as mitochondrial dysfunction, axonal degeneration, or protein misfolding within a human genetic background [137]. These cellular systems also support high-throughput drug screening under conditions closer to human physiology, thus complementing whole-animal studies and improving predictive power for clinical application. Therefore, the integration of *Drosophila*, murine, and cellular models offers a powerful complementary approach: flies for high-speed genetic analysis, mice for behavioral and systemic validation, and human cell cultures for mechanistic studies and personalized therapeutic testing. This combined strategy enhances our ability to elucidate disease mechanisms and bridge the gap between basic research and clinical translation in complex neurogenetic diseases. *Drosophila melanogaster* is an experimental model widely used for the validation of genetic mutations, thanks to its well-characterized genetics, rapid life cycle, and ease of manipulation. The genes involved in human genetic diseases are conserved in *Drosophila* allowing researchers to use this model to validate the role of these variants in a living biological context [138]. In the context of HSPs, a notable example is represented by mutations in the *KIF5A* gene (SPG10) [54]. The patient-derived p.N256S variant has been modeled in *Drosophila* through ectopic expression of the mutated human kinesin heavy chain (KHC) orthologue. This UAS- GAL4 approach allowed the reproduction of key pathological features, including impaired axonal transport, axonal swellings, and motor deficits, thus faithfully mimicking the human HSP phenotype [54]. Similarly, recently identified mutations in the *FICD* gene, responsible for a recessive form of HSP, have been investigated in *Drosophila* by generating knockout models lacking the endogenous *fic* gene [74]. These flies displayed reduced levels of Binding immunoglobulin protein (BiP), an essential for proper protein folding and stress response, increased oxidative stress, and locomotor defects, thereby highlighting the pathogenic contribution of impaired protein folding and ER stress in HSP [74]. For HA, *Drosophila* models have also provided relevant insights. Loss of the mitochondrial protein VPS13D, mutated in patients with spastic-ataxic syndromes, was studied through knockdown in flies, which exhibited abnormal mitochondrial morphology and defective axonal transport. These findings supported a mitochondrial-based mechanism underlying disease progression [119]. Taken together, these examples illustrate how *Drosophila* models, whether based on overexpression of human mutated genes or on targeted loss of endogenous orthologs, have contributed to the identification of novel disease-causing mutations, clarified the molecular pathways involved, and strengthened genotype–phenotype correlations in both HSP and HA [135]. An additional strength of the *Drosophila* model is its ability to be used in high-throughput drug screenings, enabling the identification of compounds capable of modulating the neurological defects observed in rare diseases [75]. By combining high-throughput compatibility with robust phenotypic assays, it accelerates target identification and preclinical validation, optimizing resources before vertebrate testing, *Drosophila* has been widely used for both primary screens and secondary validation of biologically active compounds [24]. In the field of HSP, 6 autophagy modulating compounds were tested in SPG15 RNAi *Drosophila* model and one of these rescued lysosomal parameters, autophagic lysosomal reformation defects and locomotor deficits [42]. In the field of ataxias, models of FRDA generated via RNAi have been employed to perform secondary drug screening. This model enabled the testing of 12 drugs that showed specificity (they did not rescue another slow-growing strain) and were not toxic at high doses. Two compounds rescued the developmental delay, and one of these also ameliorated cardiac dilation defects in adult *Drosophila* with *fxn* RNAi [138]. Another example of *Drosophila* uses in drug screening involved *fmr1*(-/-) mutants, a model for studying Fragile X Syndrome. A high-throughput approach was employed, screening approximately 2000 FDA-approved drugs. From these, 15 compounds were selected for further analysis [24]. In addition to being an excellent platform for primary screening, the fruit fly can also be effectively used in a secondary validation phase. This approach enables the rapid selection of a smaller, higher-quality subset of active compounds from a larger pool, which can then undergo medicinal chemistry optimization and subsequent testing in murine models. This strategy helps optimize resources and accelerates the overall drug discovery process. The development of *Drosophila* “avatar” models, genetically engineered to carry patient-specific mutations, represents an emerging and powerful tool in the era of precision medicine. These models allow functional validation of individual genetic variants identified in patients, offering a rapid and cost-effective strategy to assess variant pathogenicity and to test personalized therapeutic options [139]. In avatar flies, human mutations are introduced into the homologous fly gene (when conserved), or human transgenes carrying the mutant sequence are expressed in relevant tissues. These models preserve whole-organism context and are particularly useful for evaluating complex phenotypes such as locomotion, neurodegeneration, seizure susceptibility, and drug response [139]. In cancer research, the pioneering work of Ross Cagan and collaborators has demonstrated the feasibility of using *Drosophila* avatars to screen drug combinations tailored to individual patients’ genetic profiles [140]. Notably, such models have led to the identification of personalized multi-drug regimens with clinical benefit in advanced, treatment-resistant cancers. For example, in a patient with metastatic colorectal cancer harboring a *KRAS* mutation, a fly avatar enabled the discovery of a combination therapy (trametinib and zoledronate) that achieved a 45% reduction in tumor burden [141]. Beyond oncology the group led by Richard Burke used fly avatars carrying *ATP7A* and *ATP7B* mutations to uncover pathogenic mechanisms and identify candidate compounds [142]. The application of this approach to HA and HSP holds strong potential. Several *Drosophila* models have been developed that recapitulate patient-relevant aspects of these diseases. For instance, in HSP, mutations in the gene *atlastin* have been extensively studied. The in vivo effects of four pathogenic missense mutations were analyzed using two complementary approaches: CRISPR/Cas9 editing to introduce the variants into the endogenous gene, and transgenesis to generate *Drosophila* lines overexpressing *atlastin* carrying the patient-derived pathogenic variants [48]. Similarly, in FRDA, CRISPR/Cas9-mediated introduction of GAA repeat expansions into the fly *frataxin* orthologue leads to phenotypes including reduced lifespan, motor impairment, and oxidative stress [115]. Models of SCA7 have also been generated through expression of mutant *ataxin-7,* producing neurodegenerative phenotypes useful for genetic modifier screens [99]. These examples highlight the suitability of *Drosophila* avatars for dissecting the functional impact of patient-specific variants and for high-throughput in vivo drug screening.

The future of *Drosophila* “avatars” in precision medicine is particularly promising, one major avenue being the functional characterization of variants of uncertain significance (VUS) identified through patient genome sequencing [143]. Fly avatars allow rapid in vivo assessment of variant pathogenicity, offering a cost-effective approach to prioritize variants for further study in mammalian systems or patient-derived cellular models. Advances in genome editing technologies, such as CRISPR/Cas9, base editors, and prime editors, will enable increasingly precise introduction of patient-specific mutations, including point mutations, indels, and regulatory region variants. This precision will allow modeling of compound heterozygous mutations, complex alleles, or combinations of multiple patient-specific variants, expanding the ability to replicate real patient genotypes in vivo. Integration with multi-omics approaches, such as transcriptomics, proteomics, and metabolomics, can deepen the understanding of disease mechanisms in fly avatars [139]. By correlating molecular changes with phenotypic outcomes, researchers can identify biomarkers, disease pathways, and potential therapeutic targets more efficiently. Coupled with automated high-throughput behavioral and physiological assays, these approaches can reveal subtle neurobehavioral or organ-specific defects that are otherwise difficult to detect. *Drosophila* avatars also offer a powerful platform for personalized drug discovery and repurpose [144]. Large-scale chemical libraries, including FDA-approved drugs, can be screened in patient-specific fly models to identify compounds that rescue disease phenotypes. Future directions may include the combination of avatars with organ-specific modeling, such as tissue-specific expression of human mutations, optogenetic manipulation of neuronal circuits, or modeling interactions between multiple organs or cell types.

In conclusion, *Drosophila melanogaster* represents an extremely versatile and powerful model for studying rare genetic diseases, thanks to its unique combination of advanced genetic tools, low experimental costs, rapid life cycle, and high conservation of fundamental biological mechanisms with humans, making it a valuable resource for translational research and the development of targeted therapies.

## Figures and Tables

**Table 2:** Summary table of HA genes relevant to *Drosophila* research, including conserved orthologues and loci without orthologue, the genetic models developed, methodologies employed, and main phenotypes observed.

Human Genes	Protein Function	Identity and Similarity	*Drosophila* Genes	Behavior	Morphology	References
SCA1/*ATXN1*	Chromatin-binding protein acting as a transcriptional regulator; represses Notch signaling in absence of NICD by serving as CBF1 co-repressor	38% and 58%	*atx-1*	shortened lifespan and worsened motor function	degenerations in the eye, and visible phenotypes in the wing and bristles	[87,88]
SCA2/ *ATXN2*	RNA-binding protein regulating endocytosis and trafficking of EGFR; modulates actin cytoskeleton and organelle transport	24% and 36%	*atx-2*	decreased locomotion of larvae	morphological defects in the nervous system, altered cytoskeletal network, Impaired organelle transport	[89,90]
SCA3/*ATXN3*	Deubiquitinating enzyme involved in protein homeostasis maintenance, transcription, cytoskeleton regulation, myogenesis and degradation of misfolded chaperone substrates	-	no orthologue	shortened lifespan	severe and progressive adult-onset neural degeneration, compromise in vision	[29,91,92,81]
SCA5/ *SPTBN2*	protein (beta-III spectrin) is a crucial structural component of the neuronal plasma membrane, especially in Purkinje cells, where it helps stabilize glutamate transporters (EAAT4)	56% and 72%	*β-Spec*	posterior paralysis	reduced synaptic transmission, reduced synaptic terminal growth, and axonal transport deficits	[93,94,95]
SCA6/ *CACNA1A*	P/Q-type voltage-gated calcium channel α1 subunit; controls Ca2+ entry into neurons and Ca2+-dependent processes including neurotransmitter release and gene expression	43% and 54%	*cac*	shorter life span	retinal disruption	[96,97,98]
*SCA7/* *ATXN7*	Acts as a component of the SAGA (also known as STAGA) transcription coactivator-HAT complex	-	no orthologue	Impaired movement, and early lethality	neural and retinal degeneration	[19,25,99,100]
SCA8/ *ATXN8*	encodes a nearly pure polyglutamine expansion protein in the CAG direction	-	no orthologue	shortened lifespan	progressive neurodegeneration in the adult retina	[101,102]
SCA12/*PPP2R2B*	The B regulatory subunit might modulate substrate selectivity and catalytic activity and might also direct the localization of the catalytic enzyme to a particular subcellular compartment. Within the PP2A holoenzyme complex, isoform 2 is required to promote proapoptotic activity	68% and 78%	*tws*	fail to hatch, shortened life span	neurodegeneration, apoptosis, mitochondrial abnormalities	[103]
SCA13/ *KCNC3*	Voltage-gated potassium channel that plays an important role in the rapid repolarization of fast-firing brain neurons	60% and 75%	*shaw/shawl*	shortened lifespan	aberrant differentiation and polarity of photoreceptor cells, aberrant wing vein	[31,104]
SCA15/ *ITPR1*	Inositol 1,4,5-trisphosphate-gated calcium channel that, upon inositol 1,4,5-trisphosphate binding, mediates calcium release from the ER	57% and 70%	*itpr*	flight defective, locomotor behavior defective, and feeding behavior defective	neurophysiology and neuroanatomy defects	[105]
SCA17/ *TBP*	The TFIID basal transcription factor complex plays a major role in the initiation of RNA polymerase II (Pol II)-dependent transcription	58% and 63%	*tbp*	late-onset locomotor impairment and shortened lifespan	progressive neurodegeneration	[106,107,108,109]
SCA28/*AFG3L2*	Catalytic subunit of the mitochondrial m-AAA protease; regulates proteostasis of inner mitochondrial membrane proteins, essential for axonal development and neuronal survival	63% and 74%	*afg3l2*	behavioral defects, shortened lifespan, locomotor deficits	mitochondrial functional deficits and neurodegeneration	[110]
SCA31/*BEAN1*	is one of several proteins that interact with NEDD4, a member of a family of ubiquitin–protein ligases	-	no orthologue	shorter lifespan, progressive locomotor defects	severe degenerative eye morphologies, neurodegeneration progresses with age	[28,111]
SCA35/ *TGM6*	Catalyzes the cross-linking of proteins and the conjugation of polyamines to proteins	30% and 55%	*tg*	reduced the survival of models	death of primary neurons	[112]
DRPLA/*ATN1*	Transcriptional corepressor. Recruits NR2E1 to repress transcription. Promotes vascular smooth cell (VSMC) migration and orientation	23% and 31%	*gug*	shortened lifespan	eye depigmentation and retinal collapse, neural defects	[28,113,114]
FRDA/ *FXN*	mitochondrial protein which belongs to the FRATAXIN family. The protein functions in regulating mitochondrial iron transport and respiration	42% and 57%	*fh*	shortened life span, reduced climbing abilities, enhanced sensitivity to oxidative stress, cardiac defects	neuroanatomy and neurophysiology defect	[83,84,115,116]
*ATM*	Serine/threonine protein kinase which activates checkpoint signaling upon double strand breaks (DSBs), apoptosis and genotoxic stresses such as ionizing ultraviolet A light (UVA), thereby acting as a DNA damage sensor	22% and 40%	*tefu*	rough eyes, notched wings, and shorter or missing bristles	neuron and glial cell death in the adult brain, neuroblast display mitotic abnormalities	[117,118]
SCAR4/*VPS13D*	Mediates the transfer of lipids between membranes at organelle contact sites	29% and 47%	*vps13D*	reduced survival to adulthood	changes in mitochondrial morphology and impairment in mitochondrial distribution along axons	[119]
SCAR21/*SCYL1*	Regulates COPI-mediated retrograde protein traffic at the interface between the Golgi apparatus and the endoplasmic reticulum	44% and 57%	*yata*	shortened lifespan	developmental abnormalities, progressive eye vacuolization	[120]
SCAR24/*UBA5*	1-like enzyme which specifically catalyzes the first step in ufmylation	63% and 75%	*uba5*	locomotor defects, and shortened lifespan	neuroanatomical defects in larval neuromuscular junctions	[121,122]
SCAR25/*ATG5*	an E1-like activating enzyme in a ubiquitin-like conjugating system	48% and 67%	*atg5*	significant mobility defects	disruption in autophagy activity	[123]
SCAR27/*GDAP2*	Ganglioside Induced Differentiation Associated Protein 2	40% and 60%	*gdap2*	shortened lifespan, locomotor defects, and increased sensitivity to stressors	progressive neurodegeneration	[124]
SCAR32/*PRDX3*	Thiol-specific peroxidase catalyzes the reduction of hydrogen peroxide and organic hydroperoxides to water and alcohols, respectively. Plays a role in cell protection against oxidative stress by detoxifying peroxides	64% and 78%	*prx3*	reduces motor behavior		[125]
*UFM1*	Ubiquitin-like modifier which can be covalently attached via an isopeptide bond to lysine residues of substrate proteins as a monomer or a lysine-linked polymer	89% and 95%	*ufm1*	locomotor defects, and shortened lifespan	neuroanatomical defects in larval neuromuscular junctions	[126]
SCAN2/*SETX*	Probable RNA/DNA helicase involved in diverse aspects of RNA metabolism and genomic integrity. Plays a role in transcription regulation by its ability to modulate RNA Polymerase II (Pol II) binding to chromatin and through its interaction with proteins involved in transcription	21% and 36%	*setx*	affects motor behavior	morphological plasticity at neuromuscular junction (NMJ) synapses	[127,128]
SCAN3/*COA7*	required for assembly of mitochondrial respiratory chain complex I and complex IV	32% and 50%	*coa7*	reduced adult lifespan, progressive impairment in locomotive ability	shortened synaptic branches of motor neurons in larval neuromuscular junctions	[129]

## Data Availability

No new data were created or analyzed in this study.
